# Unpublished genomic data–how to share?

**DOI:** 10.1186/1471-2164-15-5

**Published:** 2014-01-14

**Authors:** Shreeya Nanda, Maria K Kowalczuk

**Affiliations:** 1 BioMed Central, 236 Gray’s Inn Road, London WC1X 8HB, UK

## Abstract

The field of genomics is often cited as the branch of biology that has led the way in data sharing. In most cases, sequencing data are made publicly available immediately after generation and often before the data generators have completed their analyses. Although the pros of such openness cannot be denied, problems can arise when unpublished genomic data are shared. In this editorial we touch on these issues and discuss the roles and responsibilities of the data generators, data users and journal editors.

## 

The past decade has seen big changes in the field of genomics, not only in terms of advances in technology, but also with regard to the views on sharing the data generated [1,2]. Open data has become the buzzword of this age. No one can deny that this openness and willingness to share genomic data, both published and (perhaps more importantly) unpublished, has resulted in remarkable progress. However, when it comes to unpublished genomic data, this openness can also leave the data generators vulnerable. The community needs to balance the benefits of data sharing against the interests of the data owners, and usually the process works well.

The genomics community has measures in place to protect the data owners–data are often released under embargoes (of varying lengths, but usually not longer than 24 months) and data owners can also publish a ‘statement of intent’, i.e. outline the specific analyses they plan to undertake, when they release the data. There are also community norms–specifically the Bermuda rules [3], and the Fort Lauderdale [4] and Toronto [5] agreements–to help researchers navigate this rather sensitive issue. However, embargoes are not indefinite and neither does it seem fair to indefinitely prohibit specific analyses. It is also worth clarifying that the agreements mentioned above are so-called gentlemen’s agreements, they are not law, and their utility depends on goodwill and communication within the community, not unlike attribution and the way scientists use citation to give credit.

The key words, as we see it, are community and communication. The researchers in the field are essentially in the same boat–they could be the data generators in one case and data users in another. Without communication the boat is likely to capsize. The data generators need to be clear in their intentions and in specifying any conditions that the data are released under, and the data users need to inform the data generators and seek permission to use the data if appropriate. Perhaps also there is a need to have enforceable guidelines in place rather than relying on gentlemen’s agreements? The US National Institutes of Health (NIH) have already taken a step in this direction and have recently released a draft policy on the sharing of genomic data [6], which, if approved, will be applicable to all researchers who receive NIH funding. The guidelines cover, amongst other topics, the issue of when to release data; for raw sequence data from non-human organisms, the specified deadline is within 6 months of submission to an approved data repository.

A question that follows is–whose responsibility is it to ensure that appropriate permission has been acquired to include the analysis of unpublished genomic data in a manuscript? Does the responsibility lie with the authors or the reviewers or with the journal editors? In our experience, such issues have usually been brought to light during the review process, but given the extensive amounts of data being generated, neither reviewers nor editors can be expected to be aware of the requirements for the use of each and every genome sequence. *BMC Genomics* has recently published a study by Zhao *et al*. [7], including an analysis of 103 fungal genomes. After publication it became apparent that some of these genomes were unpublished, and the authors had not informed the data owners of their intent of publishing an analysis of these genomes. Given this situation, we and the authors, in consultation with the data owners, agreed that a correction [8], whereby the authors would remove specific genomes from the analysis, was the appropriate way to proceed. In fact, only two of the disputed genomes were specifically under embargo, but after discussion with the data generators the authors agreed to remove from the analysis not only the embargoed genomes, but also an additional seven yet unpublished genomes.

Data generators, data users and journal editors all have a role to play in ensuring that the interests of all involved parties are protected, and as we have mentioned, the key to this is communication. We feel the ultimate responsibility should lie with the data user; it is up to them to ensure that they are aware of (and adhere to) any conditions set by the data generators. The latter could also make it easier for the data users by ensuring that the necessary information is readily available.

This is not to say that a journal has no responsibility however; a journal can increase awareness of the requirements in a field by incorporating guidance into their policies or instructions for authors. BioMed Central’s editorial policies [9] now include a section on the use of unpublished genomic data: “Authors using unpublished genomic data are expected to abide by the guidelines of the Fort Lauderdale and Toronto agreements. Based on broadly accepted scientific community standards, the key requirement for the third parties using genomic data is to contact the owners of unpublished data (i.e., the principal investigator and sequencing center) prior to undertaking their research, to advise them about their planned analyses.” A journal is also, of course, responsible for taking the appropriate action when problems such as those exemplified by this case arise. Additionally, journal editors can facilitate communication between the concerned parties and help them arrive at a mutually satisfactory solution. Finally, a journal can instigate discussion on a topic or issue by bringing them to light–as we are doing by publishing this editorial.

## Competing interests

The authors are employees of BioMed Central.

## Authors’ contributions

Both authors contributed to this editorial. Both authors read and approved the final text.

## References

[B1] Molloy JC The open knowledge foundation: open data means better science PLoS Biol 2011 15 12 e1001195 10.1371/journal.pbio.1001195 22162946 PMC3232214

[B2] Knoppers BM Harris JR Tassé AM Budin-Ljøsne I Kaye J Deschênes M Zawati MH Towards a data sharing code of conduct for international genomic research Genome Med 2011 15 7 46 10.1186/gm262 21787442 PMC3221544

[B3] Marshall E Bermuda rules: community spirit, with teeth Science 2001 15 5507 1192 10.1126/science.291.5507.1192 11233433

[B4] Sharing data from large-scale biological research projects: a system of tripartite responsibility (Wellcome Trust, 2003); available at http://www.wellcome.ac.uk/stellent/groups/corporatesite/@policy_communications/documents/web_document/wtd003207.pdf

[B5] Toronto International Data Release Workshop Authors Prepublication data sharing Nature 2009 15 7261 168 170 19741685 10.1038/461168a PMC3073843

[B6] Draft NIH genomic data sharing policy (NIH, 2013); available at http://grants.nih.gov/grants/guide/notice-files/NOT-OD-13-119.html

[B7] Zhao ZT Liu HQ Wang CF Xu JR Comparative analysis of fungal genomes reveals different plant cell wall degrading capacity in fungi BMC Genomics 2013 15 274 10.1186/1471-2164-14-274 23617724 PMC3652786

[B8] Zhao ZT Liu HQ Wang CF Xu JR Correction: comparative analysis of fungal genomes reveals different plant cell wall degrading capacity in fungi BMC Genomics 2014 15 6 10.1186/1471-2164-15-6 24422981 PMC3893384

[B9] BioMed central’s editorial policies http://www.biomedcentral.com/about/editorialpolicies

